# ^99m^Tc-labelled S-HYNIC certolizumab pegol in rheumatoid arthritis and spondyloarthritis patients: a biodistribution and dosimetry study

**DOI:** 10.1186/s13550-016-0245-0

**Published:** 2016-12-12

**Authors:** Bieke Lambert, Philippe Carron, Yves D’Asseler, Klaus Bacher, Filip Van den Bosch, Dirk Elewaut, Gust Verbruggen, Rudi Beyaert, Caroline Dumolyn, Filip De Vos

**Affiliations:** 1 Department of Radiology and Nuclear Medicine, Ghent University, De Pintelaan 185, 9000 Ghent, Belgium; 2 Department of Rheumatology, Ghent University Hospital, Ghent, Belgium; 3 Department of Basic Medical Sciences, Ghent University, Ghent, Belgium; 4 Inflammation Research Center, VIB, Ghent, Belgium; 5 Department Biomedical Molecular Biology, Ghent University, Ghent, Belgium; 6 Laboratory of Radiopharmacy, Faculty of Pharmaceutical Sciences, Ghent University, Ghent, Belgium

**Keywords:** Certolizumab pegol, Rheumatoid arthritis, Spondyloarthritis, Biodistribution, Dosimetry

## Abstract

**Background:**

Biologicals directed against tumour necrosis factor (TNF) have proven their efficacy in the treatment of spondyloarthritis and rheumatoid arthritis. We present a radiolabelling method for certolizumab pegol (CZP), a commercially available humanized Fab′-fragment directed against TNF. A biodistribution and dosimetry study was conducted.

Tc-S-HYNIC CZP was synthesized. The in vitro TNF neutralizing activity was tested by exposing L929s-cells to various concentrations 99mTc-S-HYNIC CZP and measuring TNF-induced cytotoxicity. For biodistribution and dosimetry, WB images and blood and urine sampling were performed up to 24 h pi. Cumulative activities were estimated using mono-exponential fitting, and organ doses were estimated using OLINDA/EXM. The effective dose was calculated using the International Commission on Radiological Protection 103 recommendations. The uptake of the tracer in the peripheral joints was assessed visually and semiquantitatively.

**Results:**

In vitro tests showed blocking of TNF cytotoxicity by the ^99m^Tc-S-HYNIC CZP formulation comparable to the effect obtained with the unlabelled CZP with or without the HYNIC linker. We analysed eight patients with rheumatoid arthritis or spondyloarthritis. The highest mean absorbed organ doses were recorded for kidneys, spleen, and liver: 56 (SD 7), 34 (SD 6), and 33 (SD 7) μGy/MBq. The effective dose was 6.1 (SD 0.9) mSv for a mean injected activity of 690 (SD 35) MBq. The urinary excretion was 15.1% (SD 8.1) of the IA at 22.5 h. Blood analysis yielded a distribution half-life of 1.2 h (SD 1.5) and an elimination half-life of 26.9 h (SD 2.7). Visual analysis of the scans revealed marked tracer accumulation in the clinically affected peripheral joints. In addition, there was a statistically significant higher uptake of the tracer in the swollen joints (median uptake ratio compared to background of 3.3 in rheumatoid arthritis and 2.4 in peripheral spondyloarthritis) compared to clinically negative joints (respectively 1.3 and 1.6).

**Conclusions:**

We present a radiolabelling technique for CZP, a Fab′-fragment directed against TNF and currently used as a therapeutic agent in rheumatology. An effective dose of 6.1 mSv (SD 0.9) was estimated. We confirmed the uptake of this new radiopharmaceutical in clinically affected peripheral joints.

## Background

Spondyloarthritis (SpA) and rheumatoid arthritis (RA) affect approximately 2% of the population [1, 2]. A key insight in the pathogenesis was the discovery of the important role played by proinflammatory cytokines such as tumour necrosis factor (TNF) [3, 4]. Fifteen years ago, treatment options have been revolutionized by the introduction of ‘biological agents’, including monoclonal antibodies blocking TNF. However, some patients do not or only partially respond to TNF antagonism. This might be attributable to lower levels of TNF expression, the involvement of other proinflammatory cytokines and/or the development of neutralizing antibodies against the TNF blocking agent during treatment. Therefore, on an individual patient level, proof of TNF expression in the affected joints might be helpful to optimize biological treatment directed against TNF.

Certolizumab pegol (CZP) is a Fab′ fragment derived from a humanized monoclonal antibody directed against membrane associated as well as soluble TNFα. It is commercially available as Cimzia (UCB Celltech, Slough, Berkshire, UK). In this manuscript, we describe the procedure of radiolabelling CZP and report the in vitro activity. Secondly, we document the biodistribution and dosimetric profile of this new radiopharmaceutical. Finally, we correlated the uptake of radiolabelled CZP with the findings on clinical examination in patients with peripheral joint involvement.

## Methods

### Synthesis of ^99m^Tc-S-HYNIC CZP

All preparations were carried out under aseptic conditions working in a LAF IIa cabinet.

### Derivatisation of CZP for injection with S-HYNIC

A 200-mg lyophilized CZP vial was reconstituted with water for injection (B. Braun, Melsungen AG, Germany); 100, 50 and 25 mg CZP was transferred to a Slide-A-Lyzer with cut-off of 10 kDa (Pierce Protein Research Products, Thermo scientific, Rockford, IL, USA) and dialyzed against 500 ml of a mixture of a Dulbecco’s phosphate-buffered saline (Lonza, Verviers, Belgium) and a self-prepared 0.9% *m*/*v* sodium chloride solution (Riedel-deHaën, Seelze, Germany) in 1:2 *v*/*v* ratio. Dialysis was maintained for 4 h at 2–8 °C, with the buffer refreshed after 1.5 h. Subsequently, 0.5 ml of a 8.4% sodium hydrogen carbonate (Merck, Darmstadt, Germany) solution was added to the solution followed by 10 5.0 μl portions of 1.7, 0.86 and 0.43% *m*/*v* solution of S-HYNIC (ABX GmbH, Radeberg, Germany) in dry DMSO (Merck, Darmstadt, Germany) at a pace of 1 portion/min [5]. This yielded an average of 2.8 S-HYNIC groups per CZP. After 30 min incubation at room temperature in the dark, the reaction was quenched by adding 3.0 ml cooled 0.15 M acetate buffer pH 5.0 (Merck, Darmstadt, Germany). The unreacted S-HYNIC was removed by dialyzing the reaction mixture in a Slide-A-Lyzer (cutoff of 10 kDa) overnight at 2–8 °C against 500 ml acetate buffer, which was refreshed after 1, 2 and 3 h. The solution was diluted to 40.0 ml with 0.15 M acetate buffer pH 5.0 and membrane filtered (0.22 μm). Following dispensing into 1.0 ml portions, the glass vials were stored at −80 or 2–8 °C for 3 months. Three concentrations of CZP were obtained: 2.5, 1.25 and 0.625 mg of S-HYNIC-coupled CZP. Quality control was done by determination of the protein concentration (BCA protein reagent) and the p-NBA HYNIC assay to measure the number of S-HYNIC bifunctional chelator coupled to the protein.

### Preparation of the co-ligand kit

A solution containing 4.66 mM tin(II) sulphate (Sigma Aldrich, Steinheim, Germany) and 55.81 mM tricine (Sigma Aldrich, Steinheim, Germany) dissolved in ultrapure sterile and pyrogen-free water was prepared.

### Radiolabelling with ^99m^Tc

Fifty-microliter co-ligand kit and 925 MBq (±10%) ^99m^Tc pertechnetate were consecutively added to the S-HYNIC CZP vial (2.5, 1.25 and 0.625 mg). After 15-min incubation, physiological saline was added in order to obtain a volume of 3 ml. Quality control was carried out by instant thin layer chromatography (iTLC) with SilG as stationary phase and 0.9% NaCl solution as mobile phase. For the clinical study, the 1.25 mg S-HYNIC CZP vials stored at −80 °C were used and the radiochemical yield needed to exceed 90%.

### Stability study

The impact of aggregation on the chemical stability and radiochemical yield during storage of the formulation at three different concentrations (2.5, 1.25 and 0.625 mg) was studied over a 3-month period. Aggregate formation was assessed by size-exclusion HPLC (Agilent Zorbax Diol guard column), 4 × 12.5 mm, in series with a GF450, 9.4 × 250 mm and a GF250 size exclusion analytical column, 9.4 × 250 mm (Agilent Technologies, Diegem, Belgium). The mobile phase was composed of a mixture of a 200 mM phosphate buffer pH 7.0 and ethanol 90:10 *v*/*v* (1 ml/min, 30 min). Influence on the radiochemical incorporation of ^99m^Tc was studied by iTLC as described earlier. Analyses were performed after preparation, at 2 weeks, 1 month and 3 months post production.

### In vitro activity of ^99m^Tc-S-HYNIC CZP against TNF-induced cytotoxicity

Murine fibrosarcoma TNF-sensitive L929s cells were cultured for 24 h by seeding 20,000 cells/well in 96-well plates, incubated at 37 °C with 5% CO2. The culture medium consisted of DMEM (GIBCO-BRL, 41965-062) supplemented with 10% fetal calf serum, 400 μM sodium-pyruvate (Sigma) and non-essential amino acids (Lonza). The following day, 55 μl human TNF (6.8 × 10^7^ U/ml) produced by the Protein Service facility of VIB (Ghent, Belgium) was added at various concentrations (10 dilutions starting at 300 U/ml) to the test solutions at room temperature. The following solutions of antibodies directed against TNF were tested: CZP, S-HYNIC CZP, ^99m^Tc-S-HYNIC CZP and infliximab (Remicade, Merck, Johnson & Johnson). Dilutions of 250, 50 and 10 ng/ml anti-TNF antibodies (55 μl) were preincubated with the human TNF for 45 min, as well as control samples without TNF blocking agents. Actinomycin D (Sigma, 1 mg/ml dissolved in absolute ethanol), at a final concentration of 1 μg/ml was also added. Twenty-four hours following the exposure to the test solutions, TNF cytotoxicity was measured using the MTT-test by adding 20 μl filter sterilized 3-(4,5-dimethylthiazol-2-yl)-2,5-diphenyltetrazolium bromide (MTT, Sigma, 5 mg/ml). After 4 h, 80 μl stopping solution (10% SDS, 0.01 M HCL) was added and the read out was performed by a multichannel plate reader at a wavelength of 595 nm with a reference at 655 nm. The percentage of cell survival was estimated according to the formula below:

% cell survival = (absorbance treated cells − background) / (average absorbance non treated cells − background)*100.

### Patients

The study was conducted in accordance with the Helsinki declaration and was approved by the ethical committee of the Ghent University Hospital. All patients signed an informed consent. Patient selection was limited to subjects aged 18–70 years and meeting the ‘American College of Rheumatology’ (ACR) criteria for RA [6] or the ‘Assessment of SpondyloArthritis international Society’ (ASAS) criteria for axial or peripheral SpA [7, 8]. Patients were not allowed if they had previously been treated with CZP or any other biological treatment. For more detailed information on the clinical eligibility criteria, we refer to the online detailed study protocol (EudraCT number: 2009-017998-37, http://clinicaltrials.gov/show/NCT01590966).

### Scan procedure, blood and urine sampling

All patients were scanned on the same double-headed gamma camera (BrightView, Philips Healthcare, Best, the Netherlands). First, an attenuation map was obtained from a whole-body (WB) scan using a Cobalt-57 flood source. Subsequently, the patient was injected IV with 10.6 MBq/kg ^99m^Tc-S-HYNIC CZP. The net IA was calculated following correction for rest activity in the syringe and exact time of administration (Veenstra ionization chamber VIK-202, Veenstra Instruments, the Netherlands). WB images (15 cm/min, 1024 × 512 matrix, pixel size 2.80 mm) were performed immediately following administration, at 1, 4–6 and 24 h post-injection (pi). A standard activity of approximately 5 MBq ^99m^Tc in an unshielded syringe was always in the field of view for quantification purposes. Static images (5 min, 256 × 256 matrix, pixel size 2.33 mm) of hands and feet were acquired immediately following the first WB scan, at 4–6 and 24 h.

All visualized organs (heart, lungs, liver, spleen, kidneys), as well as regions of interest (ROIs) for the WB, background and standard activity were delineated manually on the four geometric mean images, generated from the WB scans (Nuclear Diagnostics, Stockholm, Sweden). For each organ, attenuation correction factors were calculated based on measured conversion factors on our camera from ^57^Co to ^99m^Tc. These conversion factors were derived from sequential attenuation scans with a ^99m^Tc flood source and a ^57^Co flood source in the first patient. The activity measured in the urine collections between the scans was used to correct the estimated activity in the WB compartment. Cumulated activities in the WB and various organs were estimated using mono-exponential fitting and converted to time integrated activity coefficients to be processed by OLINDA/EXM 1.1 software (Stabin M. Vanderbilt University, Nashville) to estimate the absorbed organ doses. The adult male and female mathematical reference phantoms were applied. A bladder voiding interval of 4 h was included in the model. In OLINDA/EXM 1.1, effective doses (ED60) were directly calculated using ICRP 60 tissue weighting factors [9]. In order to account for the recent ICRP 103 recommendations, the effective dose was recalculated manually by combining the ICRP 103 tissue weighting factors and the organ doses from the OLINDA/EXM 1.1 output (ED103) [10]. As some organs contributing to ED103 are not listed in the OLINDA/EXM 1.1, the sum of tissue-weighting factors for the available target organs were calculated (0.913 for men and 0.922 for women). In the ED103 calculation, these factors were accounted for by scaling [11].

Urinary tracer excretion was measured in sequential urine collections at 1, 4–6 and up to 24 h pi. Total volumes were recorded. Blood sampling was performed immediately following IV tracer injection from a vein in the contralateral arm and subsequently at 1, 4–6 and at 24 h pi. ^99m^Tc activity was measured in 2 ml blood and urine samples in triplicate on a calibrated NaI(Tl) 3″ × 3″ gamma well counter (Cobra II, PerkinElmer, USA). The raw count data generated from the NaI(Tl) 3″ × 3″ detector were converted to kBq by a calibration curve. The standard samples—in the same geometry as the urine and blood samples—were prepared from a stock solution which was measured in the same calibrated dose calibrator (ionization chamber) used to measure the ^99m^Tc-HYNIC CZP syringes. Results were decay corrected and expressed as kBq/ml and as %IA.

To assess the tracer accumulation in the peripheral joints, we scored each joint visually and semiquantitatively. The latter was done by manually drawing ROIs around each joint (Hermes, Nuclear Diagnostics, Sweden). For each individual scan, a background ROI was defined within the field of view, e.g. left supraclavicular region on WB, right forearm on static images of the wrists and hands, right distal tibia for the static images depicting the ankles and feet. These results were compared with the findings on clinical examination for each assessable joint. The nuclear medicine physician reading the immunoscans and the clinician performing the clinical examination were blinded to each other’s observations. Groups were compared by Mann-Whitney *U* test using IBM SPSSv21 software (NY, USA).

## Results

### Preparation of S-HYNIC CZP

After preparation, the 2.5-, 1.25- and 0.625-mg vials contained respectively 105, 98.5 and 99.8% of the label claim for the total amount of protein indicating that no protein losses were observed during the different handling steps. The results of the p-NBA S-HYNIC incorporation test showed a respective ratio of S-HYNIC/CZP of 1.2, 1.5 and 2.0. No substantial losses were observed for the vials stored at −80 °C. In contrast, all formulations of the vials stored at 2–8 °C suffered from a decrease of the S-HYNIC/CZP ratio <0.1 after 3 months of storage.

### The influence of storage temperature on aggregation and radiochemical yield

At −80 °C, no change in the amount of aggregation was observed over time; nevertheless, the amount of aggregation (mean for the 3 formulations: 1.7%) was somewhat higher compared to the CZP blank formulation (<0.5%). At 2–8 °C, however, aggregation increased over time with the highest amount (20%) of aggregation observed for the 2.5 mg CZP formulation after 1 month of storage. All formulations stored at-80 °C had radiochemical yields >95%. For the storage at 2–8 °C, all radiochemical yields were >95% except for the 2.5 mg formulation of which the yield decreased to 55%.

### In vitro testing of the specific TNF neutralizing activity of the ^99m^Tc-S-HYNIC CZP formulation

**Fig. 1:**
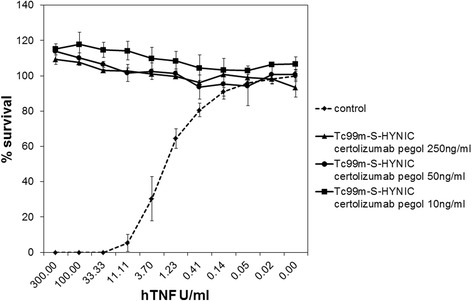
Cell survival of TNF sensitive L929s cells following exposure to 0–300 U/mL of human TNF solutions and preincubated with 250, 50 and 10 ng/ml ^99m^Tc-S-HYNIC CZP. In the control experiment, the cells are exposed to TNF but no inhibitor (^99m^Tc-S-HYNIC CZP) is added

In the samples exposed to ^99m^Tc-S-HYNIC CZP, the cytotoxic activity of TNF was completely blocked at doses as low as 10 ng/ml (Fig. 1). The TNF neutralizing effect of the ^99m^Tc-S-HYNIC CZP formulation was comparable to the effect obtained with the unlabelled CZP with or without the HYNIC linker. Infliximab, another TNF blocker that was used as a positive control, also showed good neutralizing activity, although it was less active at the doses used (Table 1).

**Table 1:** Effect on survival of TNF sensitive L929s cells following exposure to 300 U/ml human TNF and various test solutions of TNF blocking agents in the presence of 1 μg/ml Actinomycin D

	Mean % cell survival (SD)			
Tested TNF blockers at 3 concentrations	^99m^Tc-S-HYNIC CZP	S-HYNIC CZP	CZP	Infliximab
Control (no TNF blocker)	0.0 (0.0)	0.0 (0.0)	0.0 (0.0)	0.0 (0.0)
250 ng/ml	109.1 (2.6)	111.9 (0.5)	81.8 (1.5)	81.1 (3.9)
50 ng/ml	113.8 (4.2)	111.0 (2.2)	96.1 (23.2)	57.4 (1.4)
10 ng/ml	115.2 (0.6)	88.5 (0.3)	56.6 (2.8)	5.2 (0.0)

### Patient study

We analysed eight patients (four males and four females): one RA patient, two peripheral SpA patients and five axial SpA patients. The median age was 44 years (range 26–68). A mean (standard deviation, SD) activity of 690 (SD 35, range 665–728) MBq ^99m^Tc-S-HYNIC CZP (1.07 mg, SD 0.04, range 1.01–1.10) was injected. There were no adverse or clinically detectable pharmacologic effects in any of the subjects.

**Fig. 2:**
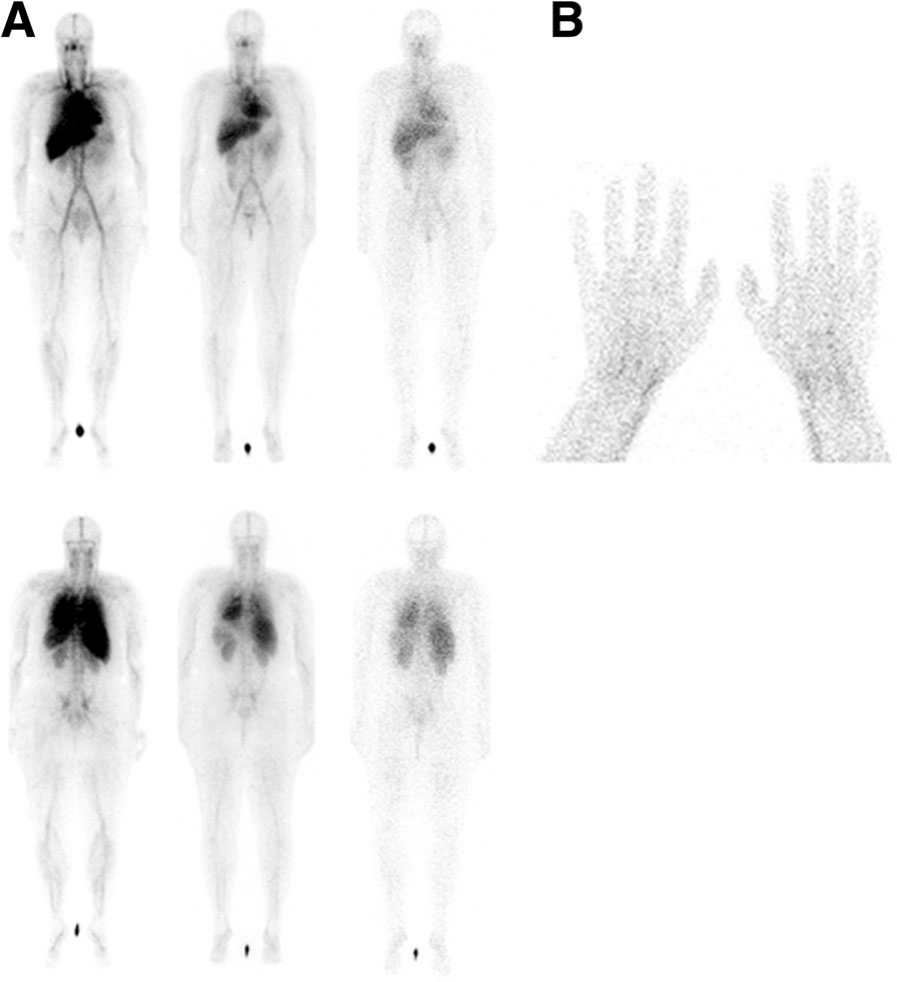
**a** WB images immediately (*left*), 5 h (*middle*) and 24 h (*right*) pi of 706 MBq ^99m^Tc-S-HYNIC CZP. **b** Static images (at 5 h pi) of the hands in a female suffering axial SpA. This patient presented with only radiographic axial involvement and had no signs of peripheral articular involvement. No enhanced tracer uptake in peripheral joints or the axial skeleton was noted

**Fig. 3:**
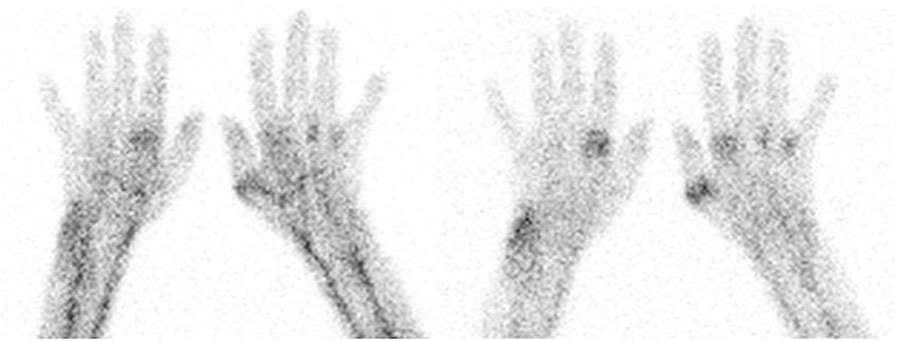
Static images approximately 15 min (*left*) and 5 h (*right*) pi of ^99m^Tc -S-HYNIC CZP in a patient suffering RA. A typical poly-articular pattern in the hand joints without distal interphalangeal involvement is observed

**Fig. 4:**
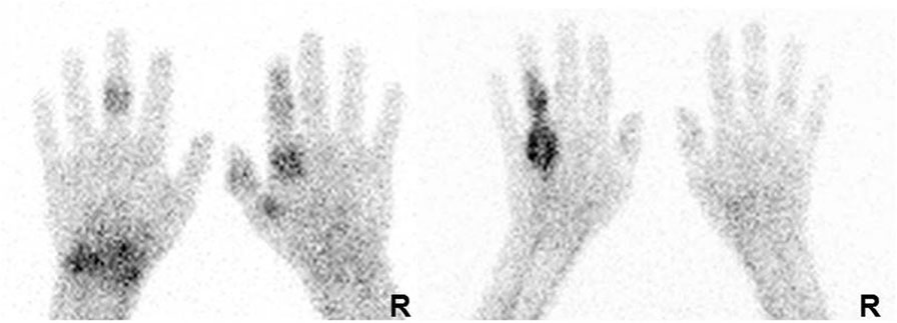
*Left*: Static images in a man suffering psoriatic arthritis, acquired approximately 5 h pi of ^99m^Tc-S-HYNIC CZP. Uptake in the metacarpophalangeal, proximal and distal interphalangeal joint of the right second digit is observed, compatible with dactylitis. *Right*: Tracer uptake 5 h pi in both the joints and the accompanying flexor tendon in a female suffering psoriatic arthritis with clinically dactylitis of the fourth left digit

On the WB images, we observed high tracer uptake in the bloodpool and liver and less pronounced uptake in the spleen and kidneys. WB scintigraphy and static images of the hands of a woman suffering axial SpA are depicted in Fig. 2 and illustrate the ‘normal peripheral distribution’ of ^99m^Tc-S-HYNIC CZP. Patients with peripheral joint involvement showed typical patterns on immunoscintigraphy with a poly-articular pattern in RA and involvement of the distal interphangeal joints, dactylitis and tenosynovitis in psoriatic arthritis (Figs. 3 and 4). The tracer uptake in the affected joints persisted over time. In the patient with RA, 16 joints showed marked tracer accumulation and 4 of these joints were clinically swollen. Out of the 50 negative joints on scintigraphy, all but 1 were also negative on clinical examination for swelling. Semiquantitative analysis yielded a median uptake ratio in swollen joints of 3.3 (range 0.6–7.5) vs 1.3 (range 0.8–5.0) in joints negative for swelling on clinical examination (*p* < 0.001 Mann-Whitney *U* test). In the 2 patients with peripheral SpA, 29 joints were visually scored positive on the immunoscan of which 20 joints were swollen on clinical assessment. Amongst the 147 joints negative on scintigraphy, 144 were also clinically classified as not swollen. Semiquantification yielded a median uptake ratio of swollen joints of 2.4 (range 1.0–6.3) vs. 1.6 (range 0.3–3.3) in non-swollen joints (*p* < 0.001 Mann-Whitney *U* test).

**Fig. 5:**
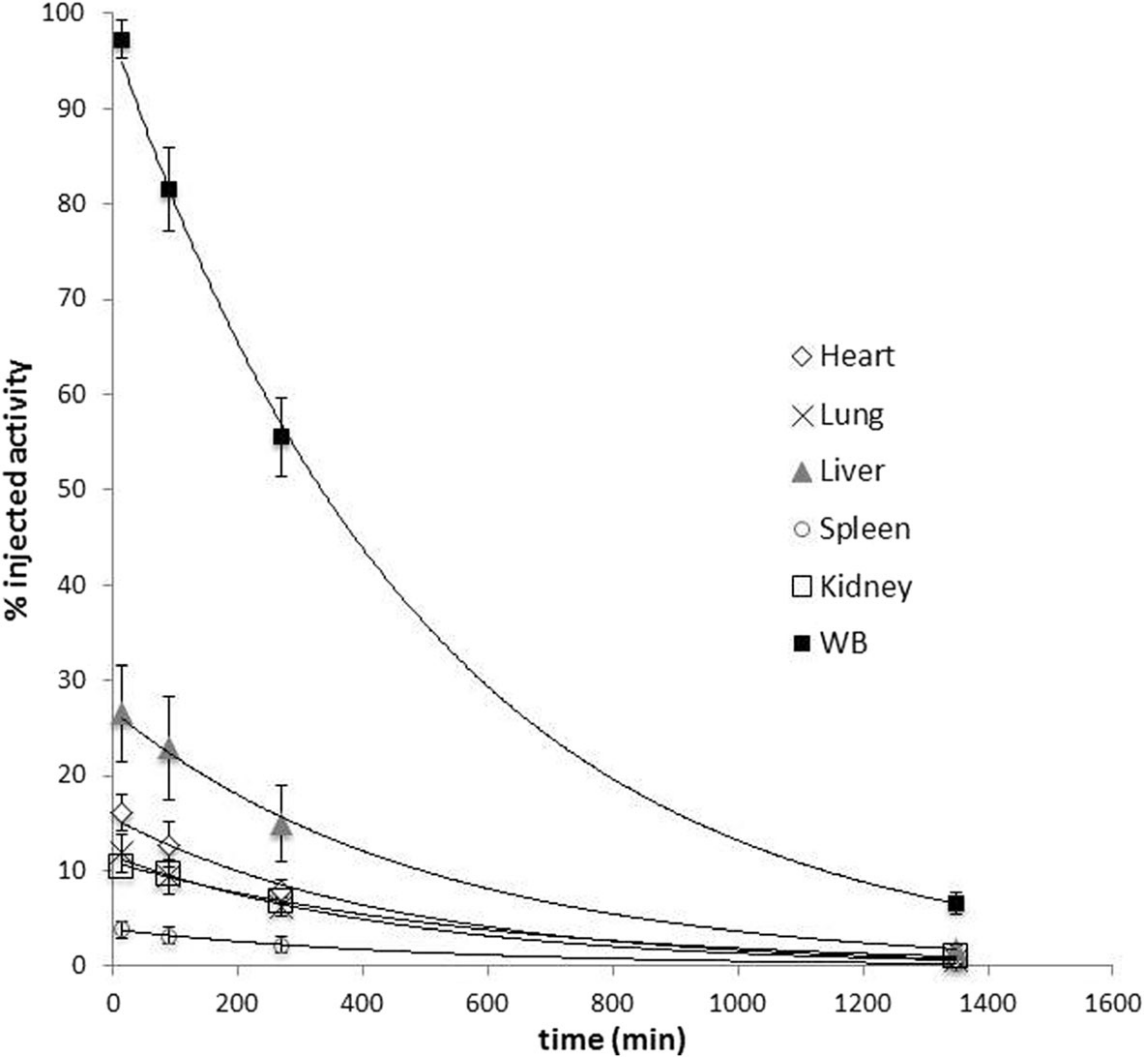
Average time-activity curves for the organs with the highest tracer uptake, as a percentage of the IA for the different organs, with standard deviations and exponential fits

**Fig. 6:**
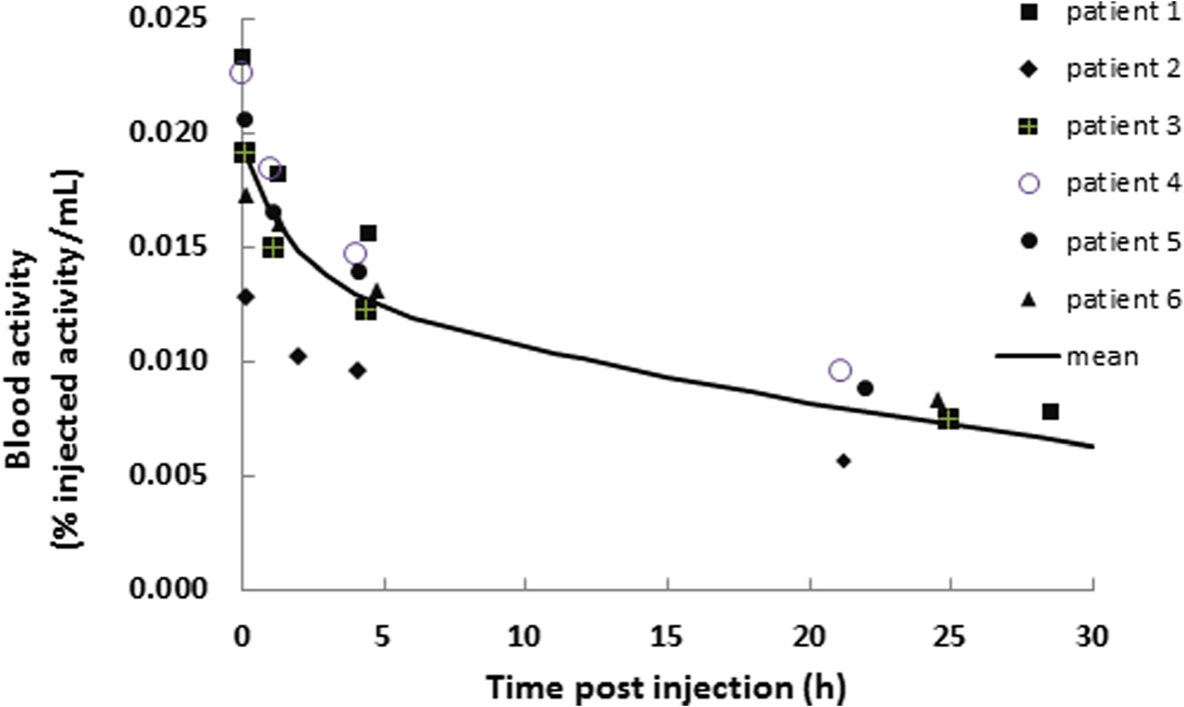
Clearance of radioactivity (kBq/ml) from the blood for all individual patients. The mean curve represents the calculated mean clearance from the blood according to a two-compartment model. Activities are decay corrected to the time of injection. A distribution half-life of 1.2 h (SD 1.5) and an elimination half-life of 26.9 h (SD 2.7) were estimated

According to our dosimetry study, the highest organ doses were attributed to the kidneys, spleen and liver with estimates of respectively 38.7 (SD 5.1) mGy, 23.4 (SD 4.3) mGy and 22.6 (SD 4.8) mGy or 56 (SD 7), 34 (SD 6) and 33 (SD 7) μGy/MBq. Table 2 summarizes our findings for the various organs included in the analysis. The estimated mean ED60 and ED103 were 8.93 (SD 1.14) μSv/MBq and 8.89 (SD 1.31) μSv/MBq, respectively. Taking into account the administered activity of 690 (SD 35) MBq, a mean ED60 and ED103 of 6.2 (SD 0.9) mSv and 6.1 (SD 1.0) mSv is obtained. Figure 5 shows the average time-activity curves as a percentage of the IA for the different organs. It can be observed from this figure that the mono-exponential fit adequately models the data. The urinary excretion was 15.1% (SD 8.1) of the IA at 22.5 h. The cumulative urinary excretion is tabulated in Table 3. The blood clearance shows a biphasic bi-exponential shape. Blood samples were fitted to a two-compartment model by the least square method. As illustrated in Fig. 6, a distribution half-life of 1.2 h (SD 1.5) and an elimination half-life of 26.9 h (SD 2.7) were estimated.

**Table 2:** Summary of the organ doses, expressed per injected MBq of ^99m^Tc-labelled S-HYNIC CZP as well as for the total mean administered activity of 690 MBq

Organs	Organ dose μGy/MBq Mean and SD		Total organ dose mGy Mean and SD	
Brain	1.3	0.5	0.9	0.3
Skin	1.8	0.3	1.2	0.2
Thyroid	2.1	0.4	1.4	0.3
Uterus	4.6	0.5	3.3	0.4
Ovaries	3.9	0.5	2.7	0.4
Testes	1.7	0.5	1.1	0.4
Breasts	3.7	0.5	2.5	0.4
Red marrow	3.8	0.4	2.6	0.3
Muscle	3.5	0.5	2.4	0.3
Small intestine	4.3	0.6	3.0	0.4
ULI wall	4.9	0.7	3.4	0.5
LLI wall	3.1	0.7	2.2	0.5
Stomach wall	6.3	1.0	4.3	0.7
Thymus	6.9	0.8	4.7	0.5
Osteogenic cells	7.4	1.4	5.1	1.0
Pancreas	10.8	1.6	7.4	1.1
Adrenals	11.2	1.7	7.7	1.2
Gallbladder wall	11.9	1.8	8.2	1.2
Lungs	18.6	2.7	12.8	1.9
Urinary bladder wall	19.9	8.3	13.7	5.8
Heart wall	30.6	5.1	21.1	3.5
Liver	32.7	6.9	22.5	4.7
Spleen	34.0	6.2	23.4	4.3
Kidneys	56.1	7.3	38.7	5.1

**Table 3:** ^99m^Tc activity measurements in urine collections at various time points

Interval p.i Mean (SD)	Cumulative urinary excretion Mean (SD) %IA
1.5 h (0.3)	4.3% (2.1)
4.5 h (0.4)	8.5% (3.2)
22.5 h (1.4)	15.1% (8.1)

## Discussion

Based on large randomized controlled trials, there is evidence for a therapeutic benefit of TNF blocking agents in RA, axial SpA and psoriatic arthritis [12–14]. Several TNF blockers have also proven their clinical value in psoriasis and inflammatory bowel disease [15, 16]. Imaging studies utilizing these biological agents could offer an individually tailored approach to pick the treatment which targets the disease best. This is in the interest of the patient who will not be exposed to unnecessary therapies. It also represents a cost saving for society, since these biologicals often involve substantial financial costs. But besides these practical potential benefits, the imaging studies open a window on various pathophysiological and pharmacokinetic aspects.

CZP differs from other TNF blockers because of its link with polyethylene glycol [17–20]. This PEGylation has been shown in animal studies to drive preferential distribution to inflamed tissue over normal tissue to a greater extent than observed with adalimumab and infliximab [20].

In the past, only few attempts were made to visualize TNF antagonists in patients [21]. So far, no radiolabelling was reported for CZP.

In a first step, we confirmed that the in vitro TNF neutralizing effect of the ^99m^Tc-S-HYNIC CZP formulation was comparable to the blocking effect observed for the unlabelled S-HYNIC CZP and the non-conjugated CZP. Considering the low ratio of radiolabelled S-HYNIC CZP to cold S-HYNIC CZP in the samples exposed to ^99m^Tc-S-HYNIC CZP, this experiment cannot serve as a direct proof of specific binding of ^99m^Tc-S-HYNIC CZP to TNF. However, the satisfying blocking effects obtained with cold S-HYNIC CZP are a strong indirect indication since it is rather the conjugation with S-HYNIC that is expected to affect the TNF affinity rather that the addition of ^99m^Tc.

In a second step, the conditions for derivatisation with S-HYNIC, storage temperature and radiolabelling of CZP were optimized. Optimal yields were obtained in storage conditions at −80 °C. Under these conditions, no cleavage of S-HYNIC from CZP was observed as indicated by the molar ratio of incorporated S-HYNIC/CZP which remained stable at 1.5 for the 1.25 mg CZP formulation. The influence of mass was demonstrated with approximately 20% of aggregates for the 2.5 mg formulation. For these reasons, the 1.25 mg CZP formulation stored at −80 °C was selected for the clinical part of the study.

The presented clinical trial reports on the first in vivo use of ^99m^Tc-S-HYNIC CZP. WB images were acquired and blood and urine samples were collected up to 24 h pi in eight patients suffering from RA or SpA. The highest organ doses were recorded for kidneys, spleen and liver. The effective dose was 6.1 mSv for a mean IA of 690 MBq. The obtained estimate for ED60 (8.9 μSv/MBq) should be viewed in the light of other tracers, such as ^99m^Tc-labelled bisphosphonates (5.7 μSv/MBq) and HIG (Human ImmunoglobulinesG) (7.0 μSv/MBq), which are—despite their limited clinical value in this specific context—often used in this patient population [22].

We observed marked tracer accumulation in the clinically affected peripheral joints, which persisted at 24 h. Barrera and coworkers published encouraging findings with radiolabelled adalimumab in 10 patients with active RA [23]. They radiolabelled this human anti-TNF monocIonal IgG1 with ^99m^Tc via S-HYNIC. They report uptake in most of the affected joints, starting within minutes after injection and persisting at 24 h. The accumulation of tracer in clinically involved joints was often a factor 2 higher compared to the clinically unaffected joints, which is concordant with our observations. However, in a clinical context of increased vascularization and capillary permeability, it is doubtful to attribute the ^99m^Tc-S-HYNIC CZP uptake predominantly to specific in vivo binding despite the encouraging in vitro assay described above.

The presented study, mainly dealing with biodistribution and dosimetry data from eight patients, was conducted in the framework of a larger clinical trial [24]. In this trial, 20 patients underwent immunoscintigraphy with ^99m^Tc-S-HYNIC CZP and were subsequently treated with CZP for 24 weeks. The expected joint involvement patterns in both peripheral and axial disease were detected by immunoscintigraphy with radiolabeled CZP: poly-articular pattern in RA, distal interphangeal involvement and dactylitis in psoriatic arthritis, and enthesitis and sacroiliitis in SpA. The probability of a joint remaining tender despite 24 weeks of CZP treatment was significantly smaller in joints with clear tracer uptake as compared to those without on baseline immunoscintigraphy.

No adverse events were recorded following injection of ^99m^Tc-S-HYNIC CZP. No clinical effect on disease activity was observed within a timeframe of 2 weeks after the scintigraphic procedure. This was within our expectations, since subtherapeutic doses of CZP were used for the imaging study.

## Conclusions

We present a radiolabeling technique for CZP, a biological agent, directed against TNF and currently indicated for treatment of moderate to severe RA, axial SpA and psoriatic arthritis. Following injection of 690 MBq ^99m^Tc-S-HYNIC CZP in eight patients, an ED103 of 6.1 (SD 0.9) mSv or 8.9 (SD 1) μSv/MBq was estimated. Urinary excretion was 15.1% of the IA at 22.5 h pi. The distribution and elimination half-life in blood was estimated to be respectively 1.2 (SD 1.5) and 26.9 h (SD 2.7). We obtained good accumulation of the tracer in the clinically affected peripheral joints of patients suffering from active RA or peripheral SpA.
